# Supplementary material for: Disrupted miRNA Biogenesis Machinery Reveals Common Molecular Pathways and Diagnostic Potential in MDS and AML
Source: Biomedicines. 2025 Dec 14;13(12):3082. doi: 10.3390/biomedicines13123082 (PMC12730432; doi:10.3390/biomedicines13123082)
Supplement: Supplementary file 1 [file biomedicines-13-03082-s001.zip › biomedicines-4003284-supplementary.pdf]

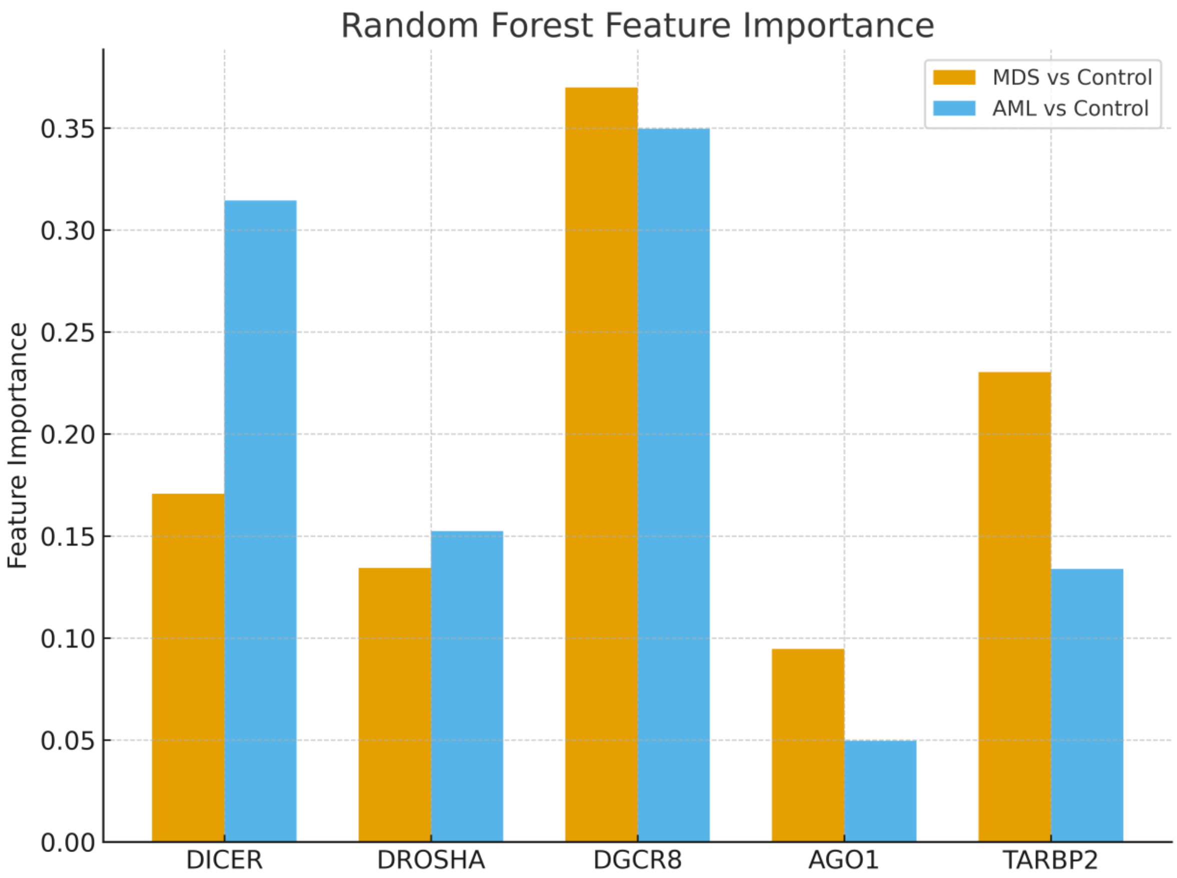

Supplementary Figure S1. Random forest feature importance analysis for miRNA biogenesis genes in MDS and AML versus controls. Bars represent the relative contribution of each gene (DICER, DROSHA, DGCR8, AGO1, and TARBP2) to the classification model. DGCR8 exhibited the strongest diagnostic relevance in both models, followed by TARBP2 and DICER in MDS, and DICER in AML.

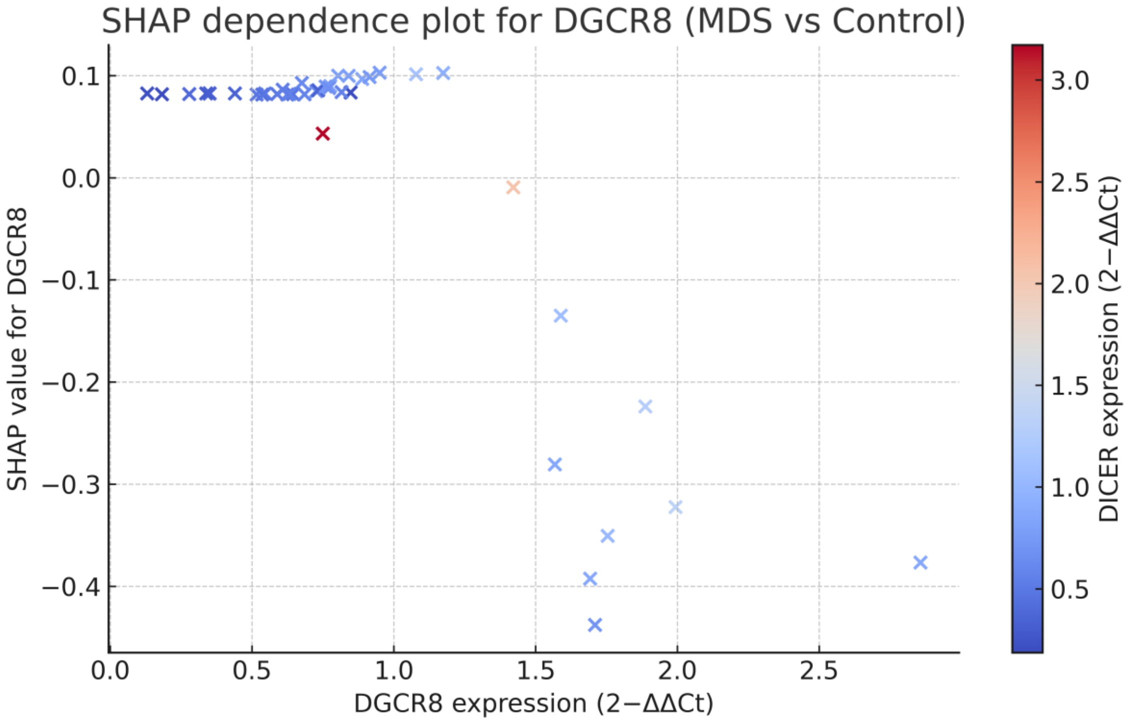

Supplementary Figure S2. SHAP dependence plot for DGCR8 in the MDS versus control model. Each point represents an individual sample. The x-axis shows DGCR8 expression (2–ΔΔCt) and the y-axis displays the corresponding SHAP value. The color scale indicates DICER expression levels, illustrating the joint influence of DGCR8 and DICER on disease probability.

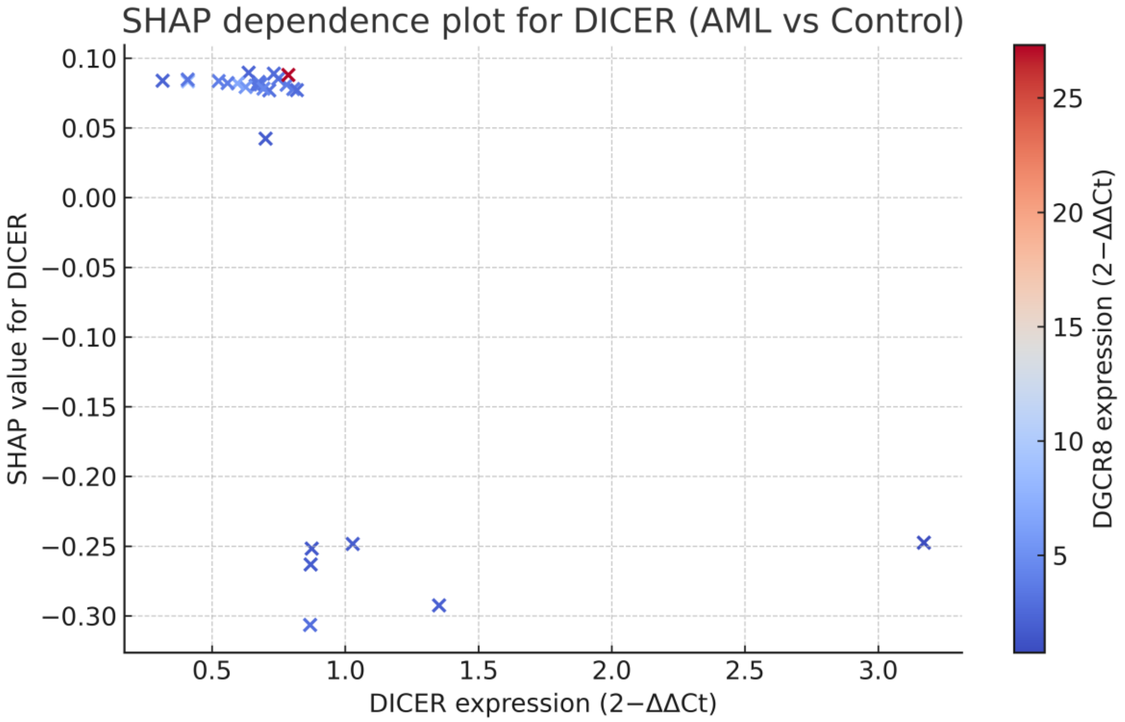

Supplementary Figure S3. SHAP dependence plot for DICER in the AML versus control model. The x-axis shows DICER expression (2–ΔΔCt) and the y-axis the associated SHAP impact. The color gradient reflects DGCR8 expression, highlighting the interaction between DICER and DGCR8 in the prediction of AML classification.
